# Hospital and Institutional News

**Published:** 1917-03-03

**Authors:** 


### HOSPITAL AND INSTITUTIONAL NEWS.

### THE PRINCE OF WALES' HOSPITAL FOR LIMBLESS SAILORS AND SOLDIERS.

It was announced at the initial meeting last Friday of the Prince of Wales' Hospital for Limbless Sailors and Soldiers at Cardiff that the institution, which was only recently established, will probably be ready for opening some time during April. The gifts towards the building and equipment fund have reached the sum of £17,500, and £4,000 has also been subscribed to the current and maintenance accounts. The Prince of Wales has become patron of the hospital and Queen Alexandra patroness. The Prime Minister (Mr. Lloyd George) and Lord Tredegar have been elected presidents, while Mrs. Lloyd George has been appointed chairman of the council and the Lord Mayor of Cardiff chairman of the executive committee. Lord Aberdare is the honorary treasurer. The appointment of Mr. Gilbert Shepherd as secretary and of Miss C. Deacon, formerly assistant matron at Roehampton, as matron, was confirmed. Lieut.-Colonel Sir Robert Jones and Lieut.-Colonel Lynn Thomas, C.B., C.M.G., have offered their services as honorary consulting orthopædic surgeons, which have been gratefully accepted by the council.

### THE GREAT CENTRAL HOTEL AS A HOSPITAL FOR OFFICERS.

Some few weeks ago the Great Central Hotel in the Marylebone Road, which had been commandeered for Army purposes some months previously, was opened as a convalescent hospital for sick and wounded officers. At the same time was inaugurated a new system of dealing with officers fit to be discharged from hospital. Until recently such officers, having been interviewed by a medical board and allotted such leave as seemed necessary, were allowed to spend that leave—once they had been discharged from hospital by the medical officer in charge—how and where they pleased. In the vast bulk of cases this meant, of course, at home or with friends; and in the enjoyment of such exercise and recreation as each man felt inclined for and capable of. A minority, however, unfortunately regarded this liberty as an excuse for licence and dissipation, and brought disgrace upon their uniform and their country by drunkenness and worse forms of debauchery. For the sins of this minority the majority has to suffer; for it has been found necessary to adopt a new and much more and restrictive system. On discharge from hospital, officers will now in many cases be sent to the Great Central Hotel, where they will remain as convalescents until fit for duty or light duty. They will then obtain a very short period of home leave, after which they must return to such duty as is allotted to them. There is no doubt that something of this sort is badly needed, and we expect to see this new experiment turn out a success.

### CHILDREN AND CHILDREN'S HOSPITALS.

The Bishop of Chichester, at the annual meeting of the Royal Alexandra Hospital for Sick Children, Brighton, commented upon the very few gifts to the institution from the children themselves—four only in the twelve months. The hint from his lordship did not fall on deaf ears. It was immediately stated on behalf of the committee of management that the board would at once consider Dr. Ridgeway's suggestion. The Bishop caused a smile when he hinted that probably, if his scheme were adopted, it would not be so well for the cinemas and sweet-shops; but these are just the agencies—particularly the cinemas—that are taking from many a home money (at least some of it) which rightly should be expended in other directions. Periodical collections should be made—they are made in some parts of the country—in all the elementary schools, because it is from these schools that the bulk of the patients come, and teachers should be looked upon to instil in the children's minds the valuable work of the hospitals. Generally among children the hospitals are taken in quite a matter-of-course sort of way, without any thought as to the source of their income and the many demands that are made upon it. The duty to subscribe regularly must be brought home to the young generation, and it is to be hoped that the Bishop of Chichester's suggestion will be adopted not only in Brighton but elsewhere.

### THE SWANSEA HOSPITAL WAR LOAN FUND.

Now that the advertisement which necessarily accompanied the issue of the War Loan has produced its fruit, and the total subscribed is known, the undertakings which individuals have entered into can be studied more impartially, and indeed with closer interest. The plan adopted for the benefit of the staff of the Swansea Hospital is this:—The committee of the hospital War Loan Fund, which undertook the purchase of £5 or multiples up to £20, offered to accept payment by deductions from salary, in the form of ten or twenty-two monthly instalments, on a scale allowing 5 per cent. per annum on each instalment from the date of its deduction. For £5 stock, ten instalments of 9s. 4d. (total £4 13s. 4d.), or twenty-two monthly instalments of 4s. 1d. (total £4 9s. 10d.), have been arranged. The instalments of course cease on December 1, 1917, or December 1, 1918, as the case may be, and when the cost is paid the subscriber receives scrip for the full amount, which then bears its 5 per cent. interest. In the event of a subscriber's death, contributions will be repaid with interest at 5 per cent. per annum. The same applies to any member of the staff who leaves, unless he or she prefers to continue the subscription. But if a subscriber discontinues his subscription, his contributions will be returned without interest. It is well to place the scheme on record, and its simplicity is its virtue. Let us turn next to its effect on the staff.

### THE WAY TO SAVE.

It needs no insurance agent to discover that the psychology of man must be studied if he is to be induced to save. Whatever their income, people run up bills faster than bank balances. Therefore very few people have the habit to save unless their saving takes the form of paying a bill. The annual or quarterly insurance premium is therefore paid, like a bill, and some people so avoid the temptation to spend instead of saving. Deductions for superannuation allowances, and weekly collections in the workshops, are similar instances to show that people will agree to save or give by spending more, rather than by spending less. In the interests of national economy the subscribers to the Victory Loan have this advantage, that saving will not be so hard to them as it remains for those who, having contracted no obligations, must choose between the difficult habit of "self-denial" and muddling on as before with nothing to show for it. Unless any member of the Swansea Hospital staff has pledged himself beyond his natural limit (which is very unlikely), we believe that every member intelligent and patriotic enough to subscribe to the War Loan on the plan set out above will have taken a step he will never regret.

### A WEEK OF GENEROUS GIFTS.

The very natural feeling on the part of hospital secretaries that all the loose money in the country has been locked up in the War Loan has been relieved by an excellent week of announcements of legacies and gifts. One of the most noteworthy is an unconditional gift of £10,000 on the part of Mr. Charles Lyle, of Abram Lyle and Sons, to Queen Mary's Hospital for the East End. The donor's son, it may be mentioned, is the chairman of the hospital committee, and it is significant, too, that the hospital received many of the sufferers in the recent explosion. The will of Lady Stewart, of Grantully Castle, Perthshire, widow of Sir Archibald Stewart, contains a long list of charitable bequests; the Royal Infirmaries, Perth and Edinburgh, will receive £1,000 each, the London Fever Hospital £1,000, Dr. Barnardo's Homes £1,000, and a number of missionary and philanthropic societies similar amounts.

### THE TOM WRIGHT NURSES' HOME AT WIGAN.

Under the will of the late Mr. T. Wright the Royal Albert Edward Infirmary, Wigan, benefits to the extent of more than eleven thousand pounds. The executors had already paid over ten thousand on account, and they have just forwarded the balance to the institution. In commemoration of this handsome bequest, the board of management has decided to name the new Nurses' Home after the benefactor; and a brass memorial tablet is to be placed at the entrance of the Home, bearing the inscription "The Tom Wright Nurses' Home." Mr. Wright was a bachelor with no near relatives; and it is of interest to note that his brother, who predeceased him, practised medicine at Wigan. It may very probably have been his brother's profession or counsel which induced Mr. Wright to devote his money to the local hospital.

### A WELL-BEFRIENDED HOSPITAL.

A donation of £150 from the president, Mrs. Williams, of Llanrumney Hall, to wipe off completely the debt upon the building, was received at the annual meeting of the board of governors of the Pontypridd Cottage Hospital. Mrs. Williams was re-elected president, and a hearty vote of thanks was tendered to her for her generous gift. The Pontypridd Hospital appears to be in a happy position all round, judging by the year's accounts. The revenue from annual subscriptions has gone up from £327 to £455, and an even more satisfactory increase has taken place in workmen's contributions, which have jumped to £614 from £487 in 1915.

### A "SAMUEL BUTLER" COT.

Major-General H. H. Lee has been able to announce the receipt, among recent donations, of one thousand guineas from the Crown Preserved Coal Company to endow a Samuel Butler cot at King Edward VII.'s Hospital, Cardiff. After a feeling of gratitude to the company, the first question which suggests itself is: Which Samuel Butler? Between Samuel Butler the wit, Samuel Butler the bishop, and Samuel Butler the philosophical writer there is bound to be some confusion, which is liable at any moment to be increased by the fact that Samuel and Butler are common names and that their conjunction is far from unusual. The adherents of either of the three great names we have mentioned must not be allowed to claim the new cot as an honour to their respective heroes until every doubt is settled, including the likelihood of a local reason for the name.

### LORD KNUTSFORD'S HUMOUR?

The "agony column" of the *Times* contained the other day a curiously worded appeal from Lord Knutsford for old linen for the London Hospital. The announcement ran as follows: "Found, outside Officers' Shell Shock Hospital, a lady's pocket-handkerchief. If owner will write to me I will tell her where she may send any others she, even uncomfortably, can do without. We need at the London Hospital every scrap of old linen we can get, and by this time this precious find has been put to better use. Address parcels Viscount Knutsford, Linen Room, London Hospital, E." Whether this rather unusual form of appeal will move many ladies to send in their pocket-handkerchiefs we are somewhat sceptical; but Lord Knutsford is such a skilled and experienced drafter that it may be assumed he thinks there is a section of the public whom this advertisement will touch. Too obvious a straining after effect, especially after a humorous effect, is a thing to be avoided as a rule in charitable appeals. Lord Knutsford may be able to make a success of semi-humorous, semi-sarcastic paragraphs such as this: but it does not follow that institutional presidents or secretaries up and down the country will be well advised to take a leaf out of his book.

### PRIVILEGED VISITING TO UNION PATIENTS.

As a result of a visitor being told that he could not see a patient, a member of a trade union, on whom a serious operation had been performed but twenty hours before, the Wolverhampton Trades Council appear to suppose that a privilege has been withdrawn. Their attitude would seem to be that society secretaries have the privilege of visiting union men at any hour, and that this supposed privilege has been denied them. Since such visits are generally made for business reasons, it is obvious that in the interests of the patients they could not possibly be privileged, and that only in exceptional cases can visits be permitted outside normal visiting hours. This simple statement of the case has been well circulated by Mr. W. H. Harper, house governor and secretary of the Wolverhampton General Hospital, and we cannot doubt that on reflection the Trades Council will endorse it.

### THE PRESENT ROUTINE OF TREATMENT AT ROCHESTER ROW.

The National Council for Combating Venereal Diseases has just published, at the modest price of twopence, a pamphlet written by Lieut.-Colonel L. W. Harrison, D.S.O., R.A.M.C., describing the present system of treatment of venereal diseases at the great military hospital in Rochester Row, Westminster, of which he is in charge. The author naturally makes no pretence to finality in his methods, which he is issuing merely as a " bulletin of progress " for the guidance of others: nor does he regard his routine as the best ideally possible, but merely as the best practicable in war conditions. It may interest those of our readers who read the description published in The Hospital (November 20, 1915, p. 161) of a great venereal clinic in the British Army sphere in France to know that the hospital there described was organised and initiated by Lieut.-Colonel Harrison, who is one of the leading authorities of the day on this special subject. With the voluntary hospitals participating in the Local Government Board schemes for combating these diseases, the publication of this authority's views in so handy and convenient a form is especially valuable; and it is to be hoped that every venereal clinic throughout the country will be provided with a copy of it. There are many novel points in Colonel Harrison's practice, some of which are not as yet to be found in the text-books.

### A NEW IDEA FROM WALTON-ON-THAMES.

The coming of peace will set the joy-bells ringing throughout the length and breadth of the country, and should throw wide open the flood-gates of generosity. Hospitals will be wise in their day and generation if they so organise matters now as to secure their proper and equitable share of the peace thank-offerings which grateful citizens will feel moved to give. We have seen no reference to this potential source of revenue in any hospital report save one—that of the Walton-on-Thames, Hersham, and Oatlands Hospital. " Should the war cease before next year," runs a passage in this report, " it is hoped that every resident of the three parishes will contribute a part of his or her peace offering to the funds of this hospital." Other institutions may copy!

### A CUSTOM WHICH HAS DISADVANTAGES.

It seems to be becoming the fashion for the presidents of hospitals and institutions which find themselves at the end of the year with only a small balance on the wrong side to liquidate the debt personally. When the president is a lady or gentleman of means and is so generously disposed, it is an easy way out of a difficulty, but the readiest solution is not always the most satisfactory in the long run. An institution which year after year finds that its income falls short of its expenditure should seek to increase its list of permanent subscriptions rather than rely on the president to find the money necessary to allow it to start the year with a clean sheet. These observations are prompted by two instances which came under our observation last week, where the debit balances of hospitals, in one case amounting to a sum of some £15, were defrayed by the president. If this custom becomes general, hospitals will lose in the long run, because energetic but not too wealthy workers will begin to decline the office of chairman or president.

### THE SECRET OF LEGACY-GETTING.

There are hospital secretaries who sit and wonder why it is that some institutions appear to be much more fortunate in the matter of receiving legacies than do others. As a rule, the secretary who allows himself mental speculation on this point is one whose institution is not among the fortunate places. It is true that there is much in the name of a hospital. No one can be surprised that the cancer hospitals receive substantial and regular help by means of legacies: the very word " cancer " has a ring of pathetic appeal about it, because everybody knows how terrible the scourge is, and also because so many families have intimate knowledge of the awful disease. Then, again, institutions for incurables are blessed with an ever-present appeal in their titles. The very helplessness and hopelessness of incurables stir the heart of the benevolent public. But a great student of human nature has said that no appeal in the world should be so strong as that made on behalf of little children. Yet, apart from the appeal of names, much can be done in the way of legacy-getting by constant, hard, and judicious work. The secretary who is keen on legacies should strive to make a friend of every subscriber. He should " follow up " every donor; he should from time to time renew his proof, or evidence, of remembrance of every subscriber and every donor. The " Christmas appeal " is looked down upon nowadays by some miscalled modern secretaries, but when infinite care is taken in its preparation it serves a double object: it is an appeal and it is a Christmas remembrance. If properly handled, the Christmas appeal should be a regular and recognised link between the institution and the supporter. One well-known institution which receives many thousands of pounds yearly in legacies has published a Christmas appeal for over sixty years; the appeals have been published every year with just one exception, and when that exception was made hundreds of letters were received from subscribers and friends asking the reason! This fact alone proves beyond a shadow of a doubt how deeply the British public loves regular and painstaking practices. No farmer expects a good crop from land that he does not fertilise, and no hospital secretary should expect good results from sources that he does not feed. Even the simple fisherman knows the value of ground-bait!

### GIFTS IN KIND TO MILITARY HOSPITALS.

An excellent idea comes from Leeds, where the Corporation greenhouses at the Canal Gardens, Roundhay Park—which are famous for their magnificent show of spring flowers in peace time—are now given over to the production of early vegetables for the sole use of the military hospitals in the city. Dwarf kidney beans from seedlings raised in pots, several varieties of peas, a few thousand cabbage lettuces, and quantities of potatoes are among the vegetables which are under process of being raised by this intensive method. So well is the cultivation advanced that it is calculated that the first crop of potatoes will be ready by the end of May, when they will be very welcome at the hospitals if the present shortage still continues. The lettuces will be fit to pick in a week or two, and the beans in a month or six weeks. The crops will be given to the hospitals for the benefit of the wounded soldiers, and when they are all cleared the twelve greenhouses will be devoted to growing tomatoes. The example of the Leeds Corporation might well be followed by other cities where the municipal greenhouses shelter nothing but flowers, and, when fresh vegetables are such an expensive item and so difficult to procure, military hospitals would look upon such assistance as indeed valuable.

### THE SHORTAGE OF QUALIFIED HOUSE OFFICERS.

On February 1 the new system of house appointments at Guy's Hospital necessitated by war conditions came into force. For some time past the claims of the military upon newly qualified men have presented an increasingly difficult problem to the hospital authorities, and they have been forced to rely upon a supply of newly qualified men not liable for service to fill up the twenty appointments hitherto deemed necessary. Under the new scheme only eleven of these are to be filled by qualified men. There are now two house physicians instead of four, two out-patient officers instead of four, and two obstetric residents instead of three; and the appointments of assistant house surgeons have been abolished. To assist these in their work four unqualified men have been appointed to the out-patient department with the title of assistant casualty officers, working on alternate days to allow them time to read for their final examinations, and similarly four assistant house physicians have been nominated to work under the two house physicians. All these officers are provided with free rooms in the College, but not with board. The appointments are made every three months. Whether or no the scheme will prove to be a success it is impossible as yet to say.

### AN ASYLUM FARM.

It is difficult to draw sound conclusions from figures in accounts without knowledge of the system on which each item is allocated, but when we read in the report of the Larbert Asylum that "the result of the year's working of the farm and garden shows a favourable balance of £503" we are ready to hope the accounts are not only accurate in figures but also represent the facts. The institution has had sufficient vegetables from the garden to supply its needs, except apparently in the case of potatoes, which did not store well and suffered from disease. Pigs, no doubt because "feed" was obtainable on the spot, were "profitable." The farm crops on the whole were good, but the fruit crop was not as good as usual. These results are encouraging, and we emphasise them in the belief that institutional gardening and farming will well bear development, since they have resources at their disposal which private persons often lack. How many institutions to-day do not make the most of their grounds for the production of vegetables?

### HOSPITAL GARDEN AMENITIES.

At some of our big London hospitals standing in outlying districts, and at many of our provincial hospitals and institutions, the gardens have hitherto been a special feature. Convalescent homes and asylums have also since their foundation made a point of surrounding themselves with pleasant gardens and grounds. But now, when everyone is told that vegetables only should be grown, the question of the garden amenities is a difficult one. Should flower seeds be purchased and sown at all this year? At one large institution (seven miles from Charing Cross) as much ground for vegetables as it will be possible to work to advantage has already been prepared for growing food, yet there is room to spare for flowers. There is no doubt that nine persons out of ten are cheered and helped by the sight of flowers growing round about. At some places the inmates of an institution are themselves able to sow and tend flowers, though they would not be able to undertake heavy spade-work, such as is necessary in the preparation of ground for potatoes and other vegetables. Any horticultural superintendent will admit that for a few shillings many pretty flowers may be grown where the ground is available and suitable. Surely, then, this dark year should be lightened by the cultivation of flowers. Whether extensive herbaceous borders and such amenities should be encouraged as heretofore is another and larger matter. If it comes to a choice between flowers without potatoes and potatoes without flowers, we take it that utilitarian principles will prevail.

### A TRIBUTE TO A DENTAL HOSPITAL.

Higher tribute to an institution could hardly be paid than that bestowed on the Birmingham Dental Hospital by the Lord Mayor of the city. Speaking at the governors' annual meeting, he observed that he wondered how they found it possible to make ends meet and carry on such a useful work with such a small subscription list. He was quite certain that no hospital money spent in Birmingham resulted in anything like the proportionate amount of good of that derived from the £600 or £700 subscribed to the Dental Hospital. The statistical return, he went on, showed that over 36,000 cases were dealt with during the year, which, although fewer than in the previous twelve months, indicated that a reduced staff had been able to do a very large amount of work. In fact, it was marvellous they had been able to do so much. A small adverse balance of £31 was reported, which is a most creditable result having regard to the increased cost of materials, drugs, etc.

### SELF-HELP IN WALES.

The gratifying announcement was made at the annual meeting of the Aberavon Hospital that every works and industry in the district—with one solitary exception—is now represented in the subscription list. This is a great achievement. The Flag Day on February 19, 1916, realised the useful sum of £600. The managing committee intimate that the day is not far distant when the question of enlarging the hospital will have to be faced.

### A HOSPITAL THAT HAS LOST TWO PRESIDENTS BY DEATH IN THREE YEARS.

A melancholy coincidence was reported at the annual meeting of the Monmouth General Hospital. For the second time within a period of three years it fell to the lot of the secretary to record the death of the president. Canon Harding was chosen to succeed as president the late Lord Llangattock, who died recently of wounds received on the Western Front. The hospital lost another good friend and benefactor during the year in the person of Mr. B. H. Deakin. The expenditure in 1916 having exceeded the income by £29 10s. 11d., the committee, as a necessary measure of economy, have decided to suspend for the duration of the war the practice of dispensing medicine to out-patients.

### AN UNEDIFYING QUARREL AT CARDIFF.

Judging from the outburst by the chairman of the health committee, there seems to be some little jealousy between the City Health Department at Cardiff and the King Edward VII. Hospital in regard to maternity and child-welfare work. In view of the forthcoming establishment by the King Edward VII. authorities of a maternity and child-welfare hospital out of the gift of £50,000 by Sir Edward Nicholl, Dr. Walford, the city medical officer, asked for authority from the health committee to send a letter to the hospital, asking for the proposals to be laid before the committee, "in view of the proposed extension of this work by the health committee and of the possibility of a further and more complete co-operation" between the two bodies. The Chairman (Dr. James Robinson) strongly supported the sending of the draft letter. It was very necessary, because at the hospital they were advertising themselves as if they had been doing this work for years, instead of the health committee, whereas they knew nothing at all about it. "There are one or two of them," continued the Chairman, "whose very existence depends on advertising themselves. I think it is time we ought to shut them up. It is our child-welfare scheme, and we want to know where we are. I am sorry to say that the co-operation the hospital is extending to us is not very much." If the health committee would back him up, concluded Dr. Robinson, he would see that they had some representation in the King Edward VII. Hospital's new scheme. The committee resolved to address a letter to the hospital as drafted by the medical officer.

### AN EXTRAVAGANT SCALE OF DIET.

A disclosure made at a meeting of the Cardiff Mental Hospital Committee last week has caused some surprise locally. A letter was received from the Board of Control asking what steps had been taken to bring the dietary for the staff into accord with the scale laid down by the Food Controller. Lieut.-Colonel Goodall admitted to the committee that no steps had so far been taken to reduce the food allowances at the hospital. The present rations, he said, varied very considerably, according to the recipient's rank. The weekly allowances to the medical officers, as compared with these suggested by Lord Devonport, were as follows:—

| | Hospital Allowance | Food Controller's Ration |
|---|---|---|
| Bread (including cake) ... | 8 lb. | 4 lb. |
| Meat (including bacon) ... | 12 lb. | 2½ lb. |
| Sugar ... ... ... ... | 1½ lb. | ¾ lb. |

The committee, considering that the present scale was far too high, decided to make an appeal to each member of the staff voluntarily to reduce this food consumption to within reasonable limits by adopting the Food Controller's standard. A special meeting is to be held next week to receive the individual replies of the staff. Meanwhile, those disposed to criticise should remember that the allowances mentioned are maximum allowances, and it may be that the actual consumption by officers at the Cardiff Mental Hospital is yet within the bounds set by the Food Controller, notwithstanding what the dietary table says.

### NO DEARTH OF HOSPITAL MOTOR DRIVERS.

All the men of the country do not appear to have been mobilised yet, notwithstanding the demands of the Army, Navy, munition, and other work of national importance, if the experience of the Bromley (Kent) Fever Hospital authorities may be taken as anything like typical. Many institutions have encountered the greatest difficulty in securing male labour as porters, attendants, and so on; but when the Bromley Hospital advertised the vacant post of motor-ambulance driver and gardener 171 applications poured in. Of course, the wages offered were good—£2 10s. a week—which leads one to believe that the smallness of the remuneration attaching to some posts connected with hospital service is often chiefly responsible for the difficulty in obtaining suitable persons to fill them.

### A MUNITION WORKER AND THE WORKHOUSE.

The Bangor and Beaumaris Board of Guardians have done well in interfering on behalf of a girl who contracted blood-poisoning at a munition works and has been moved to a workhouse infirmary. The chairman, the Rev. William Morgan, said that the girl was discharged from the hospital to which she had first been sent as soon as her funds were exhausted, and was then removed to the neighbouring workhouse infirmary. As this was a great shock to a person of her respectability he suggested that advantage should be taken of a proposal to transfer her to the Bangor Workhouse Infirmary, which the board could not suffer, by paying for her admission to the Carnarvonshire and Anglesey Infirmary, and charging the cost to the Government. The War Office is not likely to gain volunteers for National Service if it allows munition workers who fall ill as a result of their employment to enter the workhouse. Since the Guardians have lent their new infirmary to the War Office as a military hospital, and the old infirmary is practically full, they have technical as well as moral claims on the Government for the considerate and timely arrangement which they have made.

### THE V D. ACT IN VICTORIA.

The Venereal Diseases Bill, introduced to the (Victorian) Legislative Assembly in September and finally adopted, with few emendations, by the Legislative Council early in December, is now law, but not yet brought into force. Regulations are now being framed by the (State) Minister for Health. When the regulations are ready they have to receive the assent of the Governor-in-Council, and will then be put in force, probably in two or three months. The provisions of the Act seem to be effective. No special hospitals are to be established—but clinics are to be instituted at all the State-aided general hospitals; no person other than a medical man or woman—or a person directly instructed by a medical man or woman—is permitted to attend such sufferers. (This provision cuts off much lucrative custom from the chemists, who are objecting strongly.) All such sufferers are required to consult a medical practitioner, to whom they will supply their names and addresses. The medical practitioner is required to pass on the information to the inspector for the Board of Public Health, under a number; the whole medical profession to be supplied with books of numbered slips for the purpose. Thus notification and treatment are made compulsory without betrayal of the patient's identity. When a patient fails to report regularly for treatment at the times fixed by the medical practitioner, his or her name and address are immediately furnished by the doctor to the inspector of the Board of Public Health. Police magistrates are invested with powers to order the arrest and detention of all such sufferers who fail to consult medical practitioners or to attend one of the hospital clinics, those who thus fail to comply with the law are liable to a fine of £20. Those medical practitioners who fail to notify each case of these diseases treated by them are liable to a fine of £20 for the first offence and of not less than £20 nor more than £100 for a second and every subsequent offence. Unqualified persons who undertake to treat these diseases are liable to a penalty not exceeding £50, or to imprisonment for six months, with or without hard labour. Those sufferers who consider that they have been improperly arrested have the right to appeal to a police magistrate. Where children under sixteen are found to be sufferers, the onus of their cure is laid upon parents and guardians. To prevent trafficking in prescriptions, all issued to these sufferers are to be dated and signed in full by the doctor. The penalty provided for the medical practitioner who gives a false certificate of cure runs up to £50. The Act makes no provision against people contracting marriage within a certain period of their cure, the medical attendant being relied upon to warn in such cases. Regulations have to be made regarding the form of certificates of cure; the management of hospitals or clinics provided by the Health Department; the periods for which persons are to attend for treatment; measures for preventing the spread of contagion; and the fees to be allowed to medical practitioners for notification and treatment.

### THIS WEEK'S DRUG MARKET.

Compared with the rather eventful week recorded in the last report, business in the drug market during the past week has been somewhat quiet. Price changes of note have been few. A small business has been done in quinine at rather lower prices, but holders are not anxious sellers. In synthetic drugs little of importance has occurred, but barbitone is now very difficult to find in anything larger than retail quantities. The scarcity of cocaine has become more acute. Citric acid is again dearer and tartaric acid and cream of tartar still tend upwards in price. Business has been done in cascara sagrada at high prices. Honey is again dearer. Hypophosphites are very difficult to obtain in consequence of the restrictions on the small supply of raw material. Generally speaking, the difficulties in the way of business are likely to increase rather than diminish, and we may expect to see a continuance of the upward tendency of prices; this applies more especially to bulky drugs, owing to the shortage of tonnage and the steady rise in freights.

